# Caspase-7 deficiency in Chinese hamster ovary cells reduces cell proliferation and viability

**DOI:** 10.1186/s40659-020-00319-x

**Published:** 2020-11-13

**Authors:** Fatemeh Safari, Safar Farajnia, Abbas Behzad Behbahani, Habib Zarredar, Mazyar Barekati-Mowahed, Hesam Dehghani

**Affiliations:** 1 Department of Medical Biotechnology, Faculty of Advanced Medical Sciences, Tabriz University of Medical Sciences, Tabriz, Iran; 2 Biotechnology Research Center, Tabriz University of Medical Sciences, Daneshgah Ave., Tabriz, Iran; 3 Diagnostic Laboratory Sciences and Technology Research Center, School of Paramedical Sciences, Shiraz University of Medical Sciences, Shiraz, Iran; 4 Tuberculosis and Lung Diseases Research Center, Tabriz University of Medical Sciences, Tabriz, Iran; 5 Department of Physiology & Biophysics, School of Medicine, Case Western Reserve University, Cleveland, Ohio USA; 6 Department of Basic Sciences, Faculty of Veterinary Medicine, Ferdowsi University of Mashhad, Mashhad, Iran; 7 Drug Applied Research Center, Tabriz University of Medical Sciences, Tabriz, Iran

**Keywords:** CHO cells, Apoptosis, CRISPR-associated protein 9, Caspase 7, Cell proliferatio

## Abstract

**Background:**

Chinese hamster ovary (CHO) cells are the most commonly used mammalian host cell in the commercial-scale production of biopharmaceutical proteins. Modification of genes involved in apoptosis may improve the productivity of CHO cells. Executive caspases, including caspases 3 and 7, play critical roles in apoptosis. The effects of the ablation of the caspase 7 gene on proliferation and viability of CHO cells remains unknown. In this study, we applied clustered regularly interspaced short palindromic repeat (CRISPR/Cas9) to target caspase 7 gene of CHO K1 cell via all in one and homology targeted integration strategies. Consequently, the effect of caspase 7 deficiency on cell proliferation, viability, and apoptosis was studied by MTT assay and flow cytometry.

**Results:**

Findings of gel electrophoresis, western blotting, and sequencing confirmed the caspase 7 gene silencing in CHO cells (CHO-KO). Proliferation assay revealed that caspase 7 deficiency in CHO cells resulted in the reduction of proliferation in various CHO-KO clones. Besides, the disruption of caspase 7 had negative effects on cell viability in exposure with NaBu which confirmed by MTT assay. Results of flow cytometry using Anexin V/PI demonstrated that Nabu treatment (11 mM) declined the percentage of live CHO-K1 and CHO-KO cells to 70.3% and 5.79%. These results verified that the CHO-K1 cells were more resistant to apoptosis than CHO-KO, however most of CHO-KO cells undergone early apoptosis (91.9%) which seems to be a fascinating finding.

**Conclusion:**

These results reveal that caspase 7 may be involved in the cell cycle progression of CHO cells. Furthermore, it seems that targeting caspase 7 is not the ideal route as it had previously been imagined within the prevention of apoptosis but the relation between caspase 7 deficiency, cell cycle arrest, and the occurrence of early apoptosis will require more investigation.

## Background

Chinese hamster ovary (CHO) cells are the most commonly used cells for stable gene expression and producing heterologous proteins [1]. About 35% of recombinant proteins that are currently approved for human therapeutic use are produced in CHO cells [2]. Hence, the improvement of this mammalian expression system to achieve higher productivity and quality is of great industrial interest [3].

The low volumetric yield of protein is a significant challenge in the mammalian cell expression system, which is associated with a slower growth rate and high death rate of mammalian cells [4]. To respond to the market demands, cells have to be grown in large bioreactors at high densities during a prolonged period [4]. Cell culture in high density leads to environmental perturbations and cell stress due to the limitation of nutrients and oxygen, and accumulation of toxic metabolites [5].

Intense and continuous stress leads to cell death by one of the two mechanisms of passive cell death called necrosis, and apoptosis as programmed cell death. Cell death via apoptosis is identified with specific morphological characteristics and activation of a variety of cellular signaling cascades [6]. Diverse cell signaling cascades that originate as the extra- or intra-cellular stimuli can activate death-inducing pathways, downstream of caspase effectors. Caspases are divided into the inflammatory caspases and the apoptotic caspases. Apoptotic caspases are further divided into initiators. (caspases 8, 9, 10, and 12) and executors (caspases 3, 6, and 7). Initiator caspases activate executor caspases, which in turn cleave critical cellular substrates and lead to the apoptotic morphological changes [7]. Findings suggest that caspases 3 and 7 have dominant functions in apoptosis. Thereby, caspase 3 can inhibit ROS production and is an essential effector for efficient cell killing, while caspase 7 is responsible for cell detachment and ROS production [8]. Research findings show that the downregulation of caspases 3 and 7 in CHO cells promotes production while impeding apoptosis.

Various genetic engineering strategies have been established to improve the growth rate of host cells and their final yield. Thus, generating desirable genomic traits in CHO cell lines is one of the highly valuable strategies. Genome editing strategies have been traditionally performed using conventional methods such as random mutagenesis [9], homologous recombination and downregulation using siRNA [10, 11]. Nevertheless, the low frequency of desirable mutagenesis and spontaneous cleavage of chromosomal DNA led scientists to use site‐specific nucleases [12]. Site‐specific nucleases such as zinc‐finger nucleases (ZFNs) [13], transcription activator‐like effector nucleases (TALENs) [14], meganucleases [15], and the more recent clustered regularly interspaced short palindromic repeats (CRISPR)/CRISPR‐associated (Cas) system [16–19], have opened a promising window for rapid and efficient gene editing at defined genomic sites. Site‐specific nucleases employ different double-strand DNA break repair strategies including the non‐homologous end joining (NHEJ), or homology‐directed repair (HDR) [20–22].

In various studies, the CRISPR/Cas9 system has been applied to modify cell cycle-related genes, especially those involved in apoptosis. Triple knockout CHO cell lines which were attained by simultaneous disruption of FUT8, BAK, and BAX in a multiplexing setup, showed higher resistance to apoptosis [23]. Since executive caspases play significant roles in apoptosis, herein, we used CRISPR-assisted genome editing to knock out the caspase 7 gene (CASP-7). We used the multiplex CRISPR system and homology independent targeted integration (HITI) strategy to disrupt the CASP-7. Thereafter, we assessed the effects of this gene knock out on cell proliferation, viability, and apoptosis in both CHO cells.

## Results

### Application of CRISPR HITI mediated knockout strategy for targeting CASP-7

**Fig. 1:**
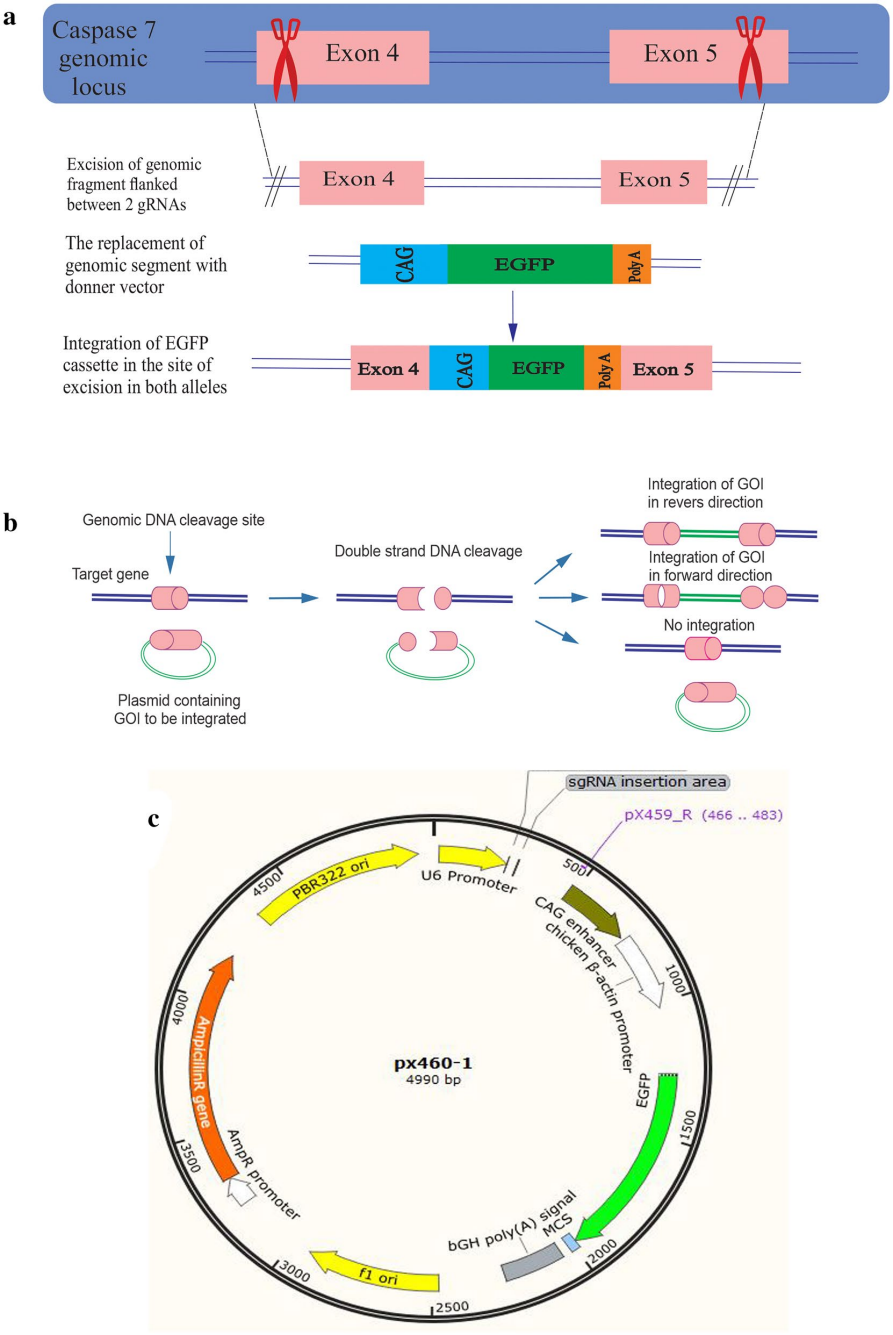
The schematic of CRISPR HITI. **a** In this strategy, two donor vectors without homology arms were utilized. These donner vectors contain sgRNA + PAM and EGFP expression cassette. In the guide of two sgRNAs, the Cas9 protein towards the exons 4 and 5 of CASP-7 at both alleles. Endonuclease function of Cas9 results in the deletion of a genomic fragment (between the exon 4 and 5) from both alleles, and the linearization of two donor vectors. The selection of cells for green color leads to the generation of caspase 7 knocked out cells. **b** The presentation of the HITI method, which uses NHEJ-mediated targeted integration in combination with Cas9 nuclease activity. If the target site remains intact or with integration in the reverse direction, DNA bears additional cleavage since forward gene insertion or gRNA can no longer bind to the target site through errors from the NHEJ repair system. **c** The schematic of PX460-1 vector which contains a single cloning site for bait gRNA and the expression cassette of the EGFP gene

**Fig. 2:**
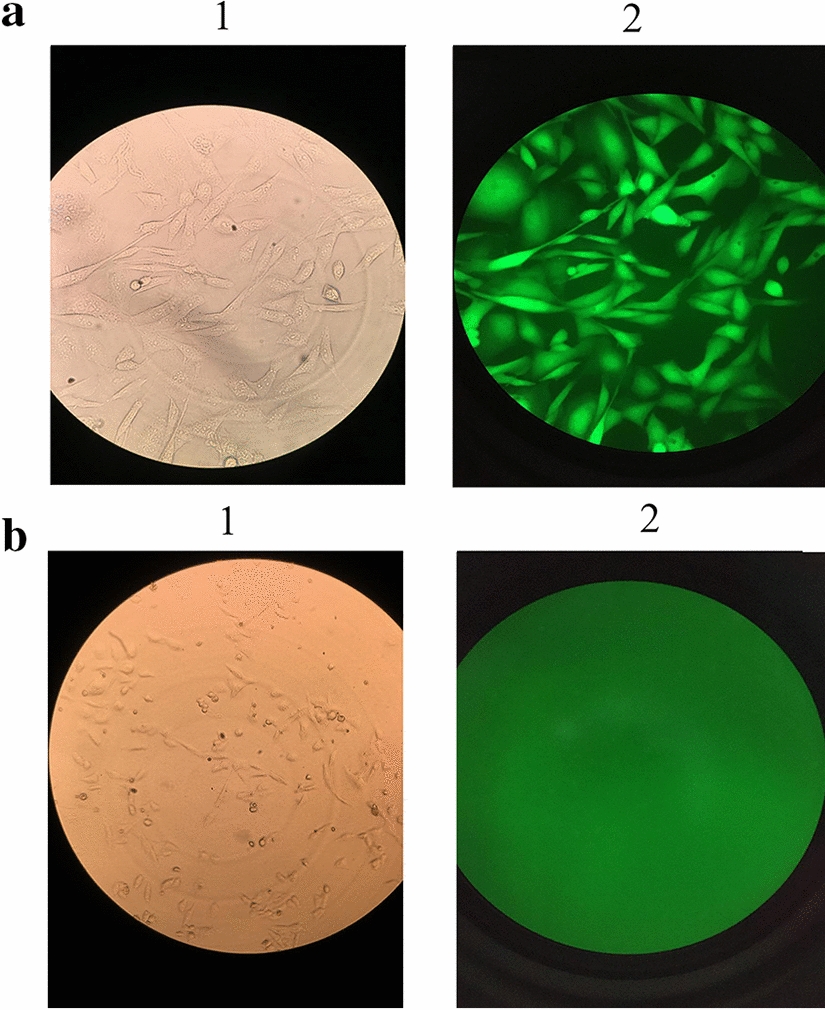
The clonal selection of EGFP expressing CHO cells. **a1** The picture of permanent EGFP expressing CHO cells (CHO-KO) on the visible light of an inverted microscope. **a2** The picture of permanent EGFP expressing CHO cells (CHO-KO) on the fluorescent light of an inverted microscope. **b1** The picture of native CHO cells (CHO-K1) on the visible light of the inverted microscope. **b2** The picture of native CHO cells (CHO-K1) on the fluorescent light of the inverted microscope

**Fig. 3:**
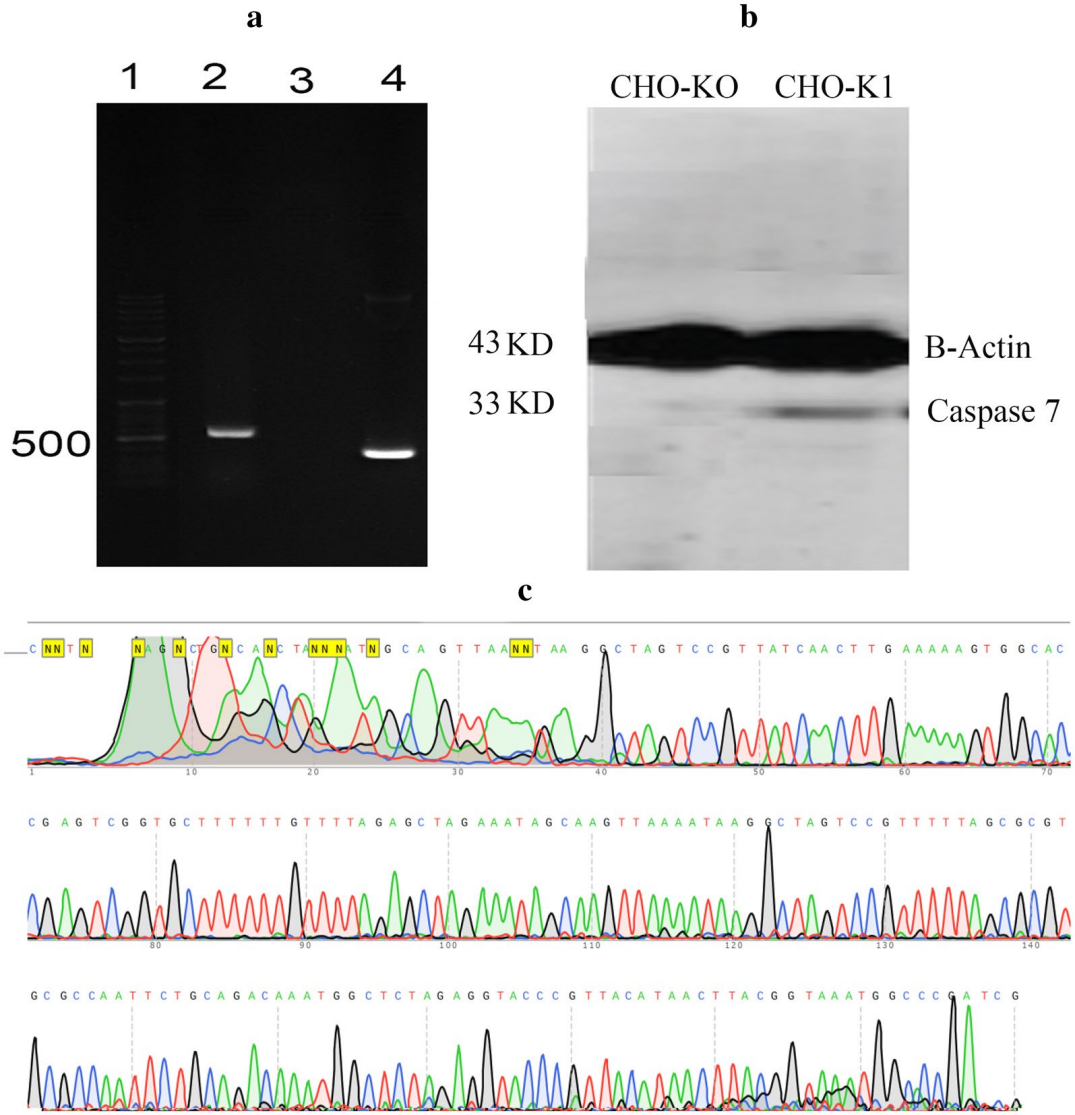
The presentations of gel electrophoresis, western blotting, and sequencing. **a** Gel electrophoresis of PCR products of both CHO-K1 (as control) and CHO-KO. Lane 1: ladder, lane 2: the PCR product of CHO-K1 using genomic primers (5′ CTGAGGAGGACCACAGCAACTC and 5′ AACTGTGGAGTAAGCGAAGAGAA), which produced a 580 bp band. Lane 3: the PCR product of CHO-KO using genomic primers (this PCR did not produce any band because the PCR product must be more than 5000 bp, which was not amplifiable by regular Taq DNA polymerase). Lane 4: a 310 bp PCR product produced by using caspase 7 genomic forward primer (5′ CTGAGGAGGACCACAGCAACTC) and a piece of PX460-1 vector (5′ CGGGCCATTTACCGTAAGTTATGTAACG) as a reverse primer. This band confirmed the integration of EGFP in the targeted site of the CHO-KO genome. **b** The presentation of western blotting. As this figure showed the genomic disruption in caspase 7 of CHO-KO cells resulted in the lack of producing of caspase 7 protein (33KD) in these cells. Beta actin was used as an endogenous control which expresses in both CHO-KO and CHO-K1 cells. **c** This picture depicts the sequencing of 310 bp fragment contained a piece of the pX460-1 vector which verified the EGFP integration in the target site of caspase 7

To target the active site of caspase 7, we aligned the sequence of this protein with the human caspase 7 sequence. Findings showed that the active site of CHO caspase 7 is flanked by exon 4 and 5. To target the active site of caspase 7, we used the “CRISPR HITI” strategy aiming for the deletion of a genomic fragment encompassing exon 4 and 5 using two sgRNAs and simultaneous insertion of a reporter (Fig. 1). After the first round of transfection, single cells were isolated by cell sorting and expanded a clone of cells that were permanently expressing EGFP (Fig. 2). The correct location of knock-in was confirmed by PCR analysis of genomic DNA. In this PCR we used a forward genomic primer and a PX460-1 reverse primer to show the integration of EGFP cassette in CASP-7. To verify the homozygosity of the clone, another PCR was performed for both wild-type (CHO-K1) and knockout cells (CHO-KO) using genomic primers (Fig. 3a). The Homozygote clone did not produce the PCR product, because the length of the integrated DNA fragment was more than 5000 bp which is not amplifiable by regular Taq polymerase, but the heterozygote clone and native CHO-K1 produced a 580 bp band. The 310 bp PCR product had been used for sequencing which confirmed the integration of EGFP (Fig. 3c). Furthermore, caspase 7 deficiency of CHO-KO cell was verified by western blotting (Fig. 3b).

### Caspase 7 deficiency negatively affected the proliferation and viability of CHO-KO cells

**Fig. 4:**
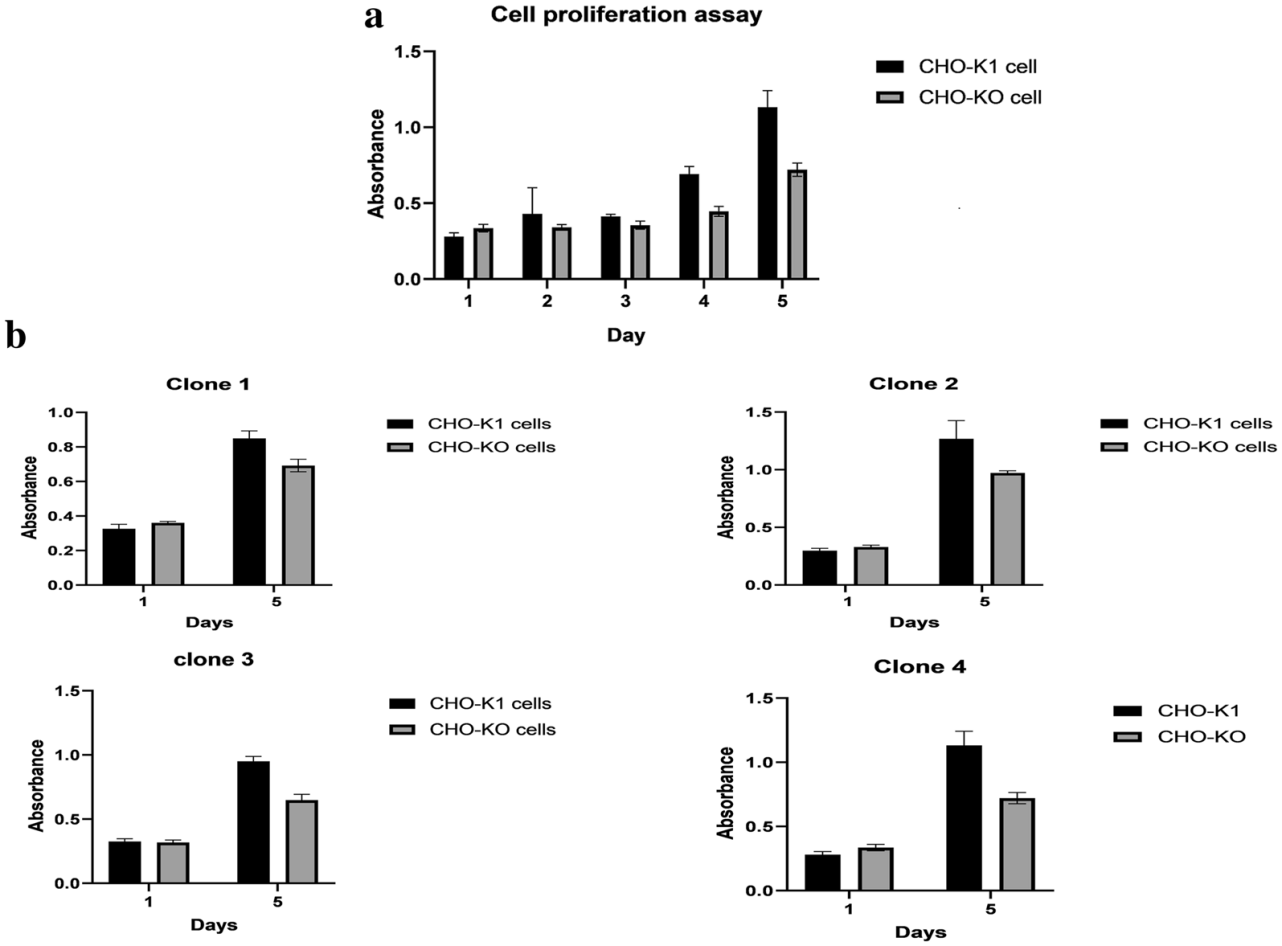
Cell proliferation assays on CHO-KO parentral and single cell clones. **a** This graph represented the results of cell proliferation assay of parentral CHO-KO clone in 1, 2, 3 and 5 days. **b** This graph demonstrated the findings of cell proliferation assay of 4 single cell clones in 1 and 5 days. These results showed that the caspase 7 disruption interfered with the cell proliferation of CHO-KO cells

Cell proliferation and cell viability are two determinant factors that affect cell productivity. To assess the CHO-KO cell growth, we used the cell proliferation assay in which the number of live cells was estimated in 5 days by measuring the absorbance of MTT for CHO-K1 and CHO-KO cells. The result of the cell proliferation assay showed that the silencing of the CASP-7 has lowered the proliferation of CHO-KO by up to 30% (p-value < 0.0001) (Fig. 4a). To confirm this difference and to eliminate the effect of CHO cell heterogeneity on cell proliferation, 4 single cell clones were isolated from the parentral CHO-KO cells and repeated the cell proliferation assays in 1 and 5 days (p-value: Clone 1 = 0.0006, clone 2 = 0.0025, clone 3 < 0.0001, clone 4 < 0.0001) (Fig. 4b). These findings confirmed the involvement of caspase 7 in cell proliferation of CHO cells.

**Fig. 5:**
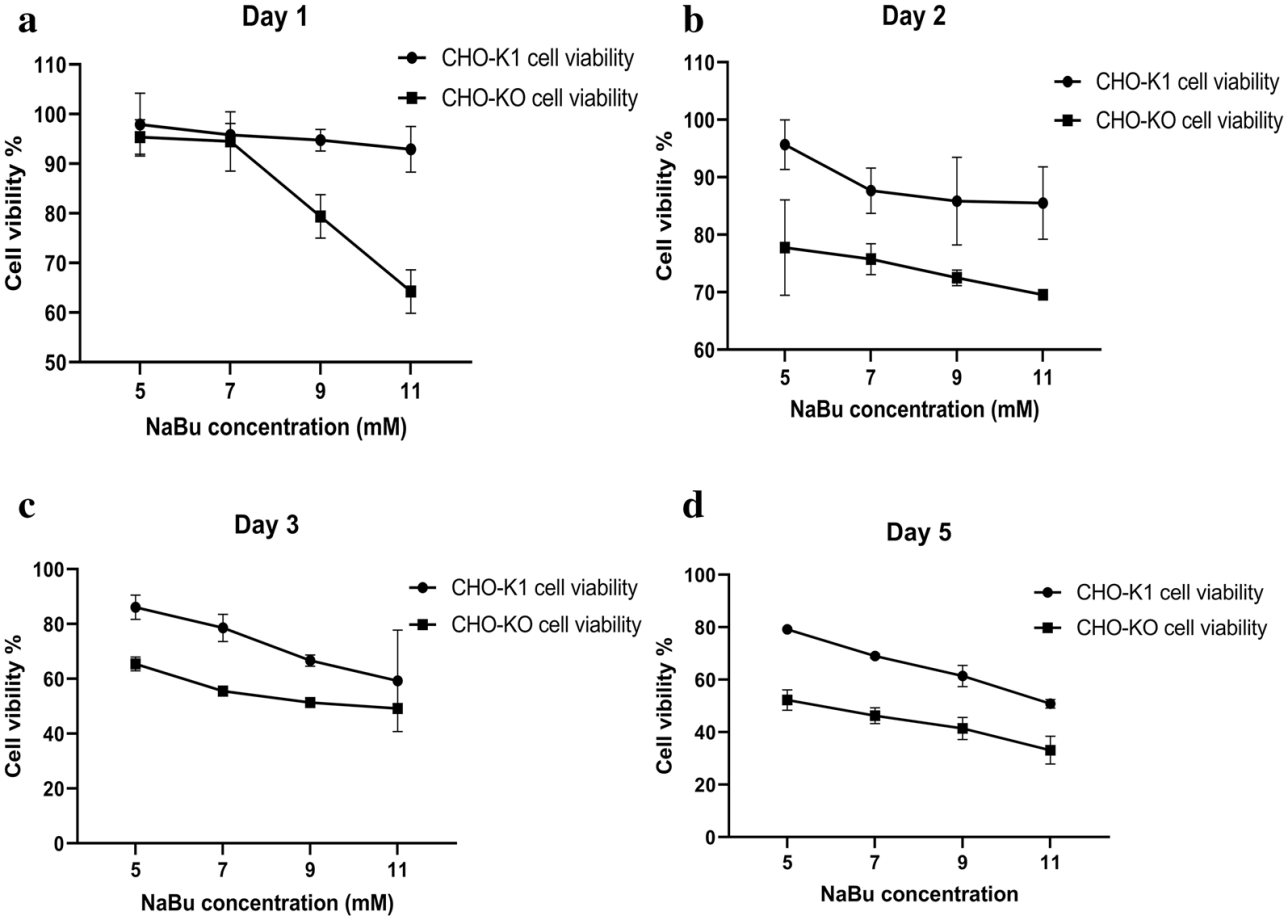
Cell viability assay. **a** This graph represented the results of cell proliferation assay in 5 days, which showed that caspase 7 disruption interfered with the cell proliferation of CHO-KO cells. **b** The assessment of CHO-K1 and CHO-KO cell viability using MTT assay (NABu was applied as an apoptosis inducer in concentrations of 5, 7, 9, and 11 mM). **a** MTT assay after 24 h exposure with NaBu. **b** MTT assay after 48 h exposure with NaBu. **c** MTT assay after 72 h exposure with NaBu. **d** MTT assay after five days of exposure with NaBu. Overly these graphs demonstrated that caspase 7 deficiency worsens the apoptosis resistance of CHO-KO in exposure with NaBu

Sodium butyrate (NaBu) is a histone deacetylase inhibitor, which increases the expression of therapeutic proteins in dose-dependent manner (0.5 to 5 mM). But at these concentrations, NaBu induces cytotoxicity due to the inhibition of cell growth and gene expression [24, 25]. Hence, to increase productivity without the irreversible cytotoxic effects, NaBu utilization must be highly controlled. Massive application of NaBu in increasing the recombinant protein productivity in CHO cells, introduces this compound as a proper apoptosis inducer in this cell line. Therefore, in this study, we used NaBu as an apoptosis inducer to investigate the apoptosis resistance of CHO-KO cells. The viability of CHO-KO cells was investigated by MTT assay using NaBu (5, 7, 9, and 11 mM) for 1, 2, 3, and 5 days (Fig. 5) which was repeated three times and in sextuplicate for each concentration of NaBu. Statistical analysis using ANOVA test showed that the silencing of caspase 7 reduced the viability of CHO-KO cells in comparison with wild type CHO-K1. (*p*-value: day 1 = 0.0001, day 2 = 0.0089, day 3 = 0.0138, day 5 = 0.0003).

Analysis of these findings showed that the reduction of CHO-KO cell viability, in addition to NaBu cytotoxicity may be due to its lower cell proliferation. Because the MTT assay only demonstrates the percentage of live cells, while the same concentration of CHO-K1 and CHO-KO cells were seeded, the lower proliferation of CHO-KO cells affected the percentage of these cells viability.

### Knockout of caspase 7 does not improve resistance to apoptosis

**Fig. 6:**
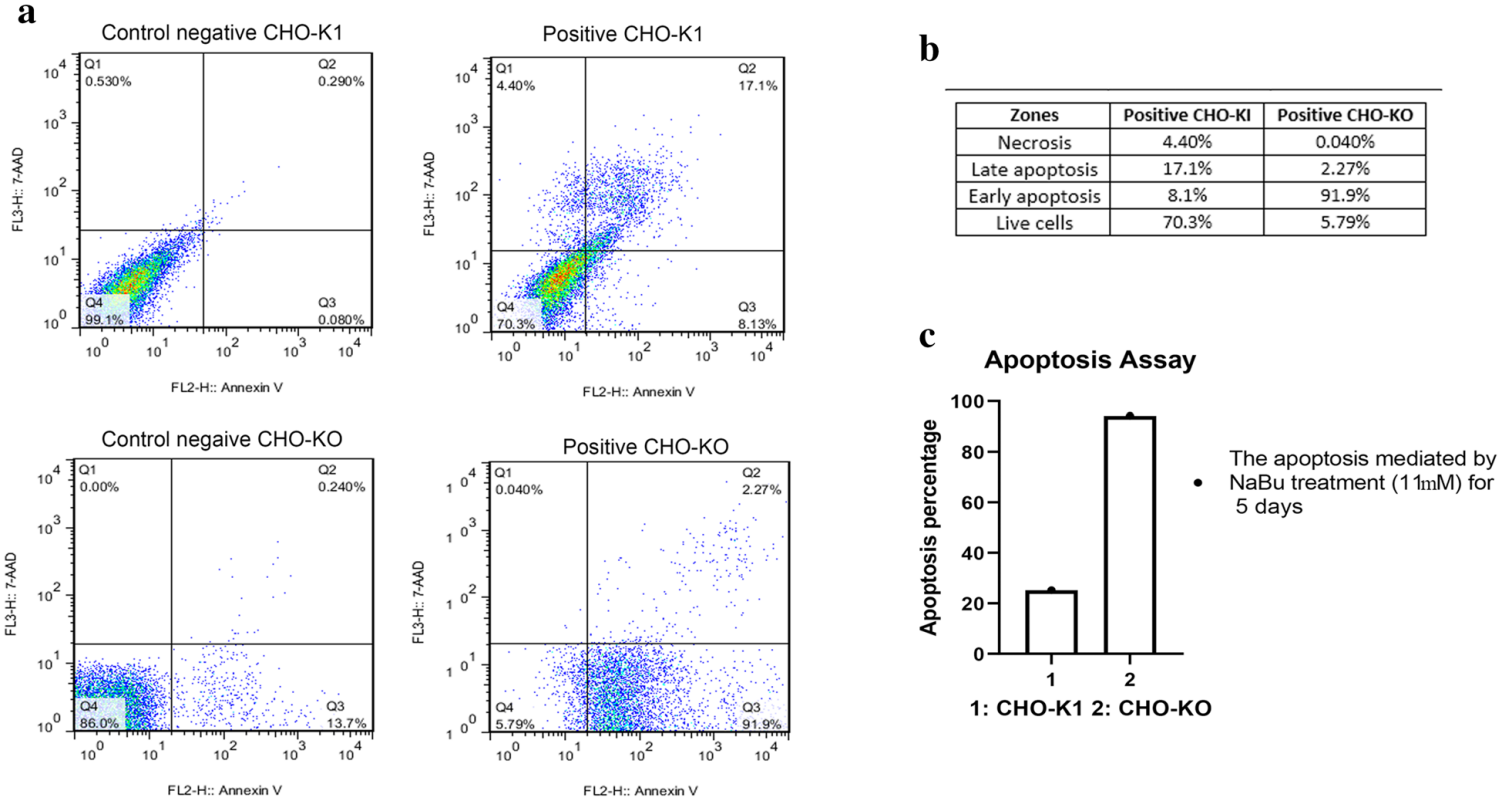
Apoptosis assay using Annexin V/PI staining. In this assay, NaBu was used as an apoptosis inducer for five days at 11 mM concentration. **a** the presentation of apoptosis assay histogram for both CHO-K1 and CHO-KO cells. **b** A table contained the cell percentage of each histogram zone for both CHO-K1 and CHO-KO cells. **c** A columnar graph that comprised the percentage of apoptosis in CHO-K1 and CHO-KO cells. Altogether, they showed that CHO-KO cell was more susceptible to apoptosis than CHO-K1 in exposure with NaBu

Flow cytometry was performed to measure the number of cells that have undergone apoptosis. Considering that Annexin V is mostly conjugated to FITC, apoptosis detection in EGFP-expressing cells can be problematic. Therefore, in this study AnnexinV/PI was used to minimize the spectral overlap between the emission profiles of FITC and EGFP fluorophores. In this assays, the negative control and NaBu-treated cells of both CHO-K1 and CHO-KO groups were assessed two times. Both cell lines were treated with NaBu for 5 days, with a concentration of 11 mM (Fig. 6). The findings of these assays are demonstrated in Fig. 5 which revealed that 94.17% of CHO-KO cells have undergone apoptosis. But only 25.2% of CHO-K1 cells were affected by apoptosis. These findings displayed that the CHO-K1 cells were more resistant to apoptosis than CHO-KO cells. The early apoptosis occurrence in 91.9% of CHO-KO cells was a fascinating event that requires more investigation.

## Discussion

CHO cells are the popular mammalian workhorse for the production of commercial therapeutically essential proteins. The development of recombinant CHO cells has become a priority in biopharmaceutical manufacture [26]. Apoptosis is one of the CHO cell challenges in the broad-scale cell culture, which affects the yield of biopharmaceutics. Hence, developing methods to impede cell death in bioreactors has been followed with a great interest [27]. In the molecular signaling pathway, programmed cell death can be initiated from three main pathways, including the death mediated by a receptor, mitochondria, or ER stress. All these three pathways are terminated to the activation of initiator and executor caspases [7].

By playing a vital role in executing caspase-dependent apoptosis, the caspases have become plausible targets for anti-apoptosis engineering. In the apoptosis, activated caspase-3 is often translocated into the nucleus, whereas active caspase-7 is associated with the ER and mitochondrial membranes. Caspases 3, 7, 8, and 9 have been targeted in CHO cells by knock-down [8, 28–33] or via the overexpression of the caspase-inhibitors such as XIAPs or CRMA [24].

In this study, the CRISPR system has been applied to knockout the CASP-7 using all in one and HITI strategies. Results of cell proliferation assay revealed that the disruption of caspase 7 expression affected the cell cycle progression and reduced cell proliferation by up to 30%.

It is the first report of the caspase 7 involvement in the CHO cell proliferation. These findings showed that besides playing a role in apoptosis, caspase 7 has noncanonical roles in proliferation and cell-cycle control. This area is less well-studied due to a few numbers of in vitro and in vivo studies which assessed the role of the caspases in cell cycle regulation. It has long been reported that cell-cycle proteins are substrates undergone proteolysis mediated by caspase 7 [34]. The examples of caspase-mediated cell-cycle proteins cleavage which causes their activation and/or translocation is Rb cleavage by caspase-3 and -7 [35]. This cleavage results in the attachment of truncated Rb to cyclin D3 and reduces E2F1 transcriptional activity [36]. Cleavage of the CHK1 (checkpoint kinase) by caspase-3 and -7 generates a truncated CHK1 with enhanced kinase activity [37, 38].

In addition, Hashimoto and his colleagues' reports showed that the silencing of caspase 7 by siRNA/shRNA resulted in cell cycle delay or arrest at the mitotic phase. Evidence of this study unveiled that during mitosis in HepG2 cells, activated caspase 3 is accumulated and also caspases 7, 8 and 9 partly become activated [39]. Also, transcriptomic studies revealed the myocyte proliferation reduction in the neonatal caspase 7 deficient heart [40]. Importantly, caspase-7 deficiency in mutant mice led to significant defects in bone formation and reduction of bone volume, mineral content, and density [41]. However it needs more investigation, but it seems that caspase 7 deficiency in CHO cells may lead to cell cycle arrest due to a delay in mitotic phase.

To assess the apoptosis resistance of knockout cells, viability assay was performed using various concentrations of NaBu. Butyrate works as a histone deacetylase inhibitor, which facilitates the relaxation of the chromatin. It consequently increases the transcription of genes and amends the gene expression in CHO cells [42]. Nevertheless, this short-chain fatty acid induces both the extrinsic and intrinsic pathways of apoptosis [43]. Results of MTT assay unveiled that the CHO-KO cells were more susceptible to apoptosis induced by NaBu than CHO-K1 cells. Also, apoptosis assay using Annexin V/PI confirmed the findings of MTT assay, which showed that knockout of caspase 7 led to undesirable effects on CHO-KO cells. We saw that CHO-K1 is more resistant than CHO-KO to apoptosis induced by NaBu (11 mM for 5 days). However, most CHO-KO cells undergone early apoptosis which may be due to the role of caspase 7 in apoptosis-related mitochondrial events. In cells undergoing stress-induced apoptosis, mitochondria may act as amplifiers of caspase activity [44, 45]. It is reported that in double knockout (caspase 3 and 7 deficient) cells, early mitochondrial events such as cytochrome c release into the cytoplasm and Bax translocation to the mitochondria were both delayed [46]. However, the process of apoptosis induced by other pathways is continued, but a delay in mitochondrial events may preserve the apoptotic cells in the early apoptosis phase. Furthermore, it seems that in the early/intermediate state of apoptosis the mitochondria may remain intact which results in functional apoptotic cells [47]. These evidences may explain the differences of MTT and flow cytometry findings by which remaining mitochondria intact at early stages of apoptosis may reduce the potential of MTT assay to detect early apoptotic cells [48].

In line with these discoveries, findings of recent meta-analysis revealed that the knock-down of caspases 3 and 7 in CHO cells generally resulted in small improvements in viable cell density (up to 40%) [33]. One of these studies reported the results with an outlier of 360% [28]. The overexpression of XIAP and CRMA as the caspase inhibitors in cultures exposed to Sindbis virus (the apoptosis inducer) displayed similar culture dynamics to controls but with a consistently lower reduction rate in viability [49]. Another group found no substantial significance in culture or titer when NaBu was used to mediate apoptosis [24]. These conflicting results have been systematically assessed, and overall, it seems that targeting caspases, or their inhibitors, is imperfect than would be expected at preventing apoptosis from a 'caspase-centric' view. This may be related to the downstream role of caspase 7, which means that cells may have already lost functionality (e.g. through MOMP) before the activation of caspases. Moreover, caspases are not involved in caspase-independent apoptosis [50].

To disrupt the CASP-7, in this study the sgRNAs targeting exon 4 and exon 5 were designed and cloned in the all in one vector. Also, the HITI strategy was employed to make the clone selection easy using donner gRNAs and vectors. Recently, HITI, as a novel genome-editing technology, has been developed, which is a combination of the NHEJ repair mechanism with the CRISPR/Cas9 system. In the HITI strategy, donor plasmids lack homology arms. Consequently, repair of the Cas9-induced genomic DSB cannot occur through the HDR pathway. The donor DNA is designed to include the Cas9 cleavage site(s) that flanks beside the donor sequence. Cas9, therefore, cleaves both the genomic target sequence and the donor plasmid, thereby generating blunt ends associated with both target and donor sequences [51]. HITI donor vectors were constructed to ensure robust gene integration only when inserted in the forward direction. If inserted in the reverse direction or is unintegrated, the DNA would undergo further cleavage by Cas9 until inserted correctly or gRNA is no longer able to bind to target sequences due to errors during NHEJ repair. The efficiency of the HITI donor vector in gene integration at target sites is approximately ten times higher than conventional HDR-mediated methods [52]. The effectiveness of HITI in non-proliferating cells (primary mouse neurons), results in gene insertion in 60% of transfected cells. Because NHEJ is active throughout the cell cycle, non-dividing cells, which are in the G0/G1 phase, also harbor NHEJ activity [53]. This study also confirmed the results of previous studies by providing successful integration of marker genes in the CHO genome. This integration resulted in the generation of caspase 7 knockout CHO cell lines.

## Conclusion

CHO cells are among the most critical host cells for the industrial production of therapeutics. Various strategies include a combination of metabolite nutrients, bioreactor process optimization, as well as the construction of high producing cell lines that are used to improve the CHO cell line. Targeting apoptosis-related genes may enhance the longevity of CHO within a culture which may subsequently improve the yield of biopharmaceutics. Executive caspases such as caspase 7 are deemed as essential proteins in the apoptosis pathway which are inducible by different stimuli. However, it has become evident that targeting these genes has propagated undesirable effects on CHO cell proliferation and viability. This demonstrates that executive caspases are imperfect targets for improving viability.

## Material and methods

### Cell lines and culture conditions

CHO cell lines were obtained from the Pasture Institute (Tehran, Iran). Cells were cultured in DMEM high-glucose (Gibco, USA) supplemented with FBS (10%), glutamine (4.05 mM), penicillin, and streptomycin (100 IU). Cultures were preserved in a humidified incubator at 37 °C with 5% CO_2_.

### sgRNAs, plasmids and cloning

In order to efficiently target the CASP-7 by knocking out/in, CRISPR guide sequences were selected using CRISPy software. To this end, designing was based on targeting exons with a high efficient (high on-target score) and high specific (low off-target effects) sgRNAs. In this study, sgRNAs were chosen for targeting the active site of the CASP-7, which flanks in exons 4 and 5. Sequences for sgRNA oligos can be found in Table 1. For the silencing CASP-7, we combined the CRISPR multiplex system and HITI strategy. Plasmids for the multiplex CRISPR/Cas9 vectors include pX330S-2 (Plasmid #58778, addgene, USA) and pX330A-1×2 (Plasmid #58766, addgene, USA), and the vector used for HITI strategy was pX460-1 which contains U6 promoter-sgRNA insertion site-sgRNA scaffold and CAG promoter-enhanced GFP (EGFP)-bovine growth hormone polyadenylation signal (gifted by Dena Zist company, Mashhad, Iran).

**Table 1:** The sequence of gRNAs and primers

Oligoes	Caspase 7 target site	sequences
Exon 4	agatggcgtgacgccaataa	Fw: CACCgAGATGGCGTGACGCCAATAA Rev: AAACTTATTGGCGTCACGCCATCT
Exon 4 + PAM	agatggcgtgacgccaataaagg	Fw: caccgCCTTTATTGGCGTCACGCCATCT Rev: aaacAGATGGCGTGACGCCAATAAAGGc
Exon 5	gatacgctttaggcatgccg	Fw: CACCGATACGCTTTAGGCATGCCG Rev: AAACCGGCATGCCTAAAGCGTATC
Exon 5 + PAM	gatacgctttaggcatgccgagg	Fw: caccCCTCGGCATGCCTAAAGCGTATC Rev:aaacGATACGCTTTAGGCATGCCGAGG
PX-460-1 primers	–	Fwd: GCCTTTTGCTGGCCTTTTGCTC Rev:CGGGCCATTTACCGTAAGTTATGTAACG
Genomic primers	–	Fwd: CTGAGGAGGACCACAGCAACTC Rev: AACTGTGGAGTAAGCGAAGAGAA

As previously described, the gRNAs targeting exons 4 and 5 were cloned in a single pX330A-1×2 by using a golden gate assembly kit [54] and HITI gRNAs, which contained PAM sequence (Table 1) were cloned in pX460-1 vectors [52].

### Transfection, single cell cloning and detection of CRISPR mediated knockout clone by PCR and sequencing

CHO cells were co-transfected with pX330A-1×2 and pX460-1 plasmids using transfectimin as a transfection reagent based on the manufacture manual. Passing a week and after several passages, the GFP positive cells were sorted by FACS (BD) Cell Sorter. The permanent GFP expressing single-cell clones were grown and screened via PCR for caspase 7 KO detection.

### Western blotting

In order to confirm the knockout of CASP-7 in CHO cell line, SDS-PAGE electrophoresis followed by western blotting was performed. CHO-K1 and CHO-KO cells (1 * 10^6^/culture flask [75 cm^2^]) were treated with NaBu for 5 days with the concentration of 11 mM/mL. Cells were harvested in amount of 5 × 10^6^ by tripsinization and lysed with RIPA buffer and sonication. Proteins obtained from cell lysates were dissected by 8% SDS-polyacrylamide gel electrophoresis (PAGE) and transferred to PVDF membranes (Millipore, Bedford, MA). Caspase 7 was detected using anti-Caspase-7 antibody (Abcam, Cat Nom: ab32522), respectively. Beta actin was used as an internal control that has a relatively uniform level of expression among different cells.

### Cell viability (MTT) and cell proliferation Assay

The clone 4 of CHO-KO and native CHO-K1 cells were respectively plated in 96 wells plates with a concentration of 5 × 10^3^ per well. The plates were kept in an incubator, and the cells were incubated for 16–24 h in standard drug-free growth medium to adhere to the surface of the plates. After the attachment, cells were exposed to NaBu at a designated concentration as 5, 7, 9, and 11 mM, respectively. The plates were put back to the incubator and kept for 1, 2, 3, and 5 days at 37 °C. After each respected day of incubation, the medium from each well was entirely removed and replaced with 100 µL of fresh free culture medium containing 10 µL of 12 mM MTT stock solution. After 4 h of incubation, MTT media was replaced by 100 µL of DMSO following mixing thoroughly using an orbit plate shaker. The plates were then incubating for additionally 10 min, then the absorbance was read at 540 and 680 nm reference wavelengths. To assess the cell proliferation, parentral CHO-KO cells and four single cell clones isolated from this cell line were seeded in 96 well plates in the mentioned concentration, and MTT assay was performed in days 1 and 5 (CHO-K1 single cell clones were used as control). Both these assays were repreated three times and in sextuplicate manner.

### Assessment of apoptosis by Annexin /PI staining

This assay was used to measure the number of cells that have undergone apoptosis. In short, 0.5 × 10^6^ cells were seeded in 6 well plates and treated with NaBu (11 mM) for 5 days. Cells were detached by trypsin and put in a polystyrene 75 × 12 mm tube, washed with 1 mL ice-cold Annexin V binding buffer (10 mM HEPES, 140 mM NaCl, 2.5 mM CaCl_2_ in dH_2_O, pH 7.4), centrifuged, and re-suspended in 100 μL binding buffer. 2.5 μL of Annexin V was added per tube and incubated for 15 min in the fridge. 400 μL of binding buffer was further added into the tube after incubation. Finally, 5 μL of 7AAD (20 μg/mL stock) was added to each tube 45 s before the flow cytometric analysis. This assay was repeated two times.

## Data Availability

The datasets used and/or analyzed during the current study are available from the corresponding author on reasonable request.

## Abbreviations

CHO: Chinese hamster ovary
ZFNs: Zinc-finger nucleases
TALENs: Transcription activator-like effector nucleases
CRISPR: Clustered regularly interspaced short palindromic repeats
Cas: CRISPR-associated protein
NHEJ: Non-homologous end joining
HDR: Homology-directed repair
HITI: Homology independent targeted integration
EGFP: Enhanced green fluorescent protein
NaBu: Sodium butyrate
DSB: Double-strand DNA break
